# *Ncl* Synchronously Regulates Na^+^, K^+^, and Cl^**−**^ in Soybean and Greatly Increases the Grain Yield in Saline Field Conditions

**DOI:** 10.1038/srep19147

**Published:** 2016-01-08

**Authors:** Tuyen Duc Do, Huatao Chen, Vu Thi Thu Hien, Aladdin Hamwieh, Tetsuya Yamada, Tadashi Sato, Yongliang Yan, Hua Cong, Mariko Shono, Kazuhiro Suenaga, Donghe Xu

**Affiliations:** 1 Japan International Research Center for Agricultural Sciences (JIRCAS), Tsukuba, Ibaraki, 305-8686, Japan; 2 Cuulong Delta Rice Research Institute (CLRRI), Thoilai, Cantho, Vietnam; 3 Institute of Vegetable Crops, Jiangsu Academy of Agricultural Sciences, Nanjing, Jiangsu, China; 4 Genetic Engineering Division, Agricultural Genetics Institute, Hanoi, Vietnam; 5 International Center for Agricultural Research in the Dry Areas (ICARDA), Cairo, Egypt; 6 Graduate School of Agriculture, Hokkaido University, Sapporo, Hokkaido, Japan; 7 Graduate School of Life Sciences, Tohoku University, Sendai, Miyagi, Japan; 8 Institute of Crop Germplasm Resources, Xinjiang Academy of Agricultural Sciences, Urumqi, Xinjiang, China

## Abstract

Salt stress inhibits soybean growth and reduces gain yield. Genetic improvement of salt tolerance is essential for sustainable soybean production in saline areas. In this study, we isolated a gene (*Ncl*) that could synchronously regulate the transport and accumulation of Na^+^, K^+^, and Cl^−^ from a Brazilian soybean cultivar FT-Abyara using map-based cloning strategy. Higher expression of the salt tolerance gene *Ncl* in the root resulted in lower accumulations of Na^+^, K^+^, and Cl^−^ in the shoot under salt stress. Transfer of *Ncl* with the *Agrobacterium*-mediated transformation method into a soybean cultivar Kariyutaka significantly enhanced its salt tolerance. Introgression of the tolerance allele into soybean cultivar Jackson, using DNA marker-assisted selection (MAS), produced an improved salt tolerance line. *Ncl* could increase soybean grain yield by 3.6–5.5 times in saline field conditions. Using *Ncl* in soybean breeding through gene transfer or MAS would contribute to sustainable soybean production in saline-prone areas.

Soybean [*Glycine max* (L.) Merr.] is the world’s primary crop source for protein and oil. The total world soybean production is 268.0 million metric tons in 2012, providing 68% of world protein meal consumption and 28% world vegetable oil consumption (www.soystats.com). Soybean cultivation is conducted in a wide range of environments and is exposed to many biotic and abiotic stresses that influence the sustainability of soybean production. Soybean is generally regarded as a salt-sensitive crop compared with other major crops such as wheat, rice, and cotton. Salt stress inhibits soybean germination and plant growth^1^,^2^, nodule formation^3^, and seed yield^4^.

Genetic variation for salt tolerance has been described in soybean^1^,^4^,^5^,^6^, The salt tolerance of partial soybean germplasm conserved in the GRIN, USDA has been evaluated. One hundred and fifty one germplasms, such as Lee, Lee 68, and S-100, were identified to be salt-tolerant, whereas 413 germplasms were designated as susceptible to salt reaction (www.ars-grin.gov/npgs/searchgrin.html). Salt tolerance variation was also observed in wild soybean [*Glycine soja* Sieb. & Zucc.] and some wild soybean accessions were identified to be salt-tolerant^7^,^8^,^9^,^10^,^11^,^12^. The high level of variation in soybean germplasm, including wild and cultivated soybean species, suggest that genetic improvement of salt tolerance is feasible.

The heredity of salt tolerance in soybean was previously analyzed as a quality trait using the classical genetics approach^13^,^14^, and the gene symbols *Ncl* and *ncl* were proposed as the dominant for tolerance and the recessive for sensitive, respectively^13^. The recent development of soybean molecular maps provides a promising approach in identifying genes associated with traits of interest in soybean^15^,^16^. Several quantitative trait loci (QTLs) for salt tolerance were reported in different germplasms^8^,^10^,^17^,^18^,^19^. Of these, a major QTL for salt tolerance was constantly detected on soybean chromosome 3 (Linkage group N) in different populations^8^,^10^,^17^,^19^. The QTL on chromosome 3 is likely to be the *Ncl* locus based on pedigree tracing^17^. The salt tolerance QTL, in which the tolerant allele was from a wild soybean accession PI 483463, was recently mapped within a 658-kb region between SSR03_1335 and SSR_1359 on chromosome 3^10^. The 658-kb region contained 80 annotated genes, including two genes (*Glyma03g32890* and *Glyma03g32900*) belonging to the sodium/hydrogen exchanger family. Using the whole-genome sequencing approach, a candidate causal gene, *GmCHX1*, a counterpart of *Glyma03g32900* in Williams 82, was identified in a wild soybean accession W05^12^. *Glyma03g32900* (*GmSALT3*), which associated with limiting the accumulation of sodium ion, was also identified as a candidate causal gene underlying the QTL on chromosome 3 from a Chinese commercial soybean cultivar, Tiefeng 8, by using fine mapping method^20^.

Functional analyses of sodium (Na^+^) and potassium (K^+^) transporters have been extensively conducted because of their roles in both nutrition and salt tolerance^21^,^22^,^23^,^24^,^25^,^26^,^27^. Several candidates for the cation antiporter associated with salt tolerance such as *GmCHX1*^12^, *GmSALT3*^20^, *GmCAX1*^28^, *GmNHX1*^29^, *GmPIP*^30^, and *GmHKT1*^31^ have been reported in soybean. In contrast, mechanisms of chloride (Cl^−^) transport in plants are poorly understood, despite the importance of minimizing Cl^−^ toxicity for salt tolerance. Control of Cl^−^ transport and Cl^−^ “exclusion” from shoots is correlated with salt tolerance in soybean^1^,^7^,^11^,^13^,^32^ and other legumes *Trifolium*^33^, Medicago^34^, and Lotus^35^ as well as in other crops, such as wheat^36^; however, no gene that regulates the transport and accumulation of Cl^−^ in soybean has been reported.

Map-based cloning or positional cloning strategies have been successfully used in the isolation of genes underlying QTLs of environmental stress tolerant traits such as salt^25^, submergence^37^, and phosphorus deficiency^38^ tolerances in crops. In this study, we isolated a QTL for salt tolerance using the map-based cloning strategy from a from a Brazilian soybean cultivar FT-Abyara to facilitate its use in breeding. Our results showed that the salt tolerance gene could synchronously regulate the transport and accumulation of Na^+^, K^+^, and Cl^−^ in soybean. The near isogenic lines (NILs) carrying the tolerant allele of the QTL could increase soybean grain yield by 3.6–5.5 times in saline field conditions.

## Results

### Map-based cloning of the salt tolerance QTL

**Figure 1:**
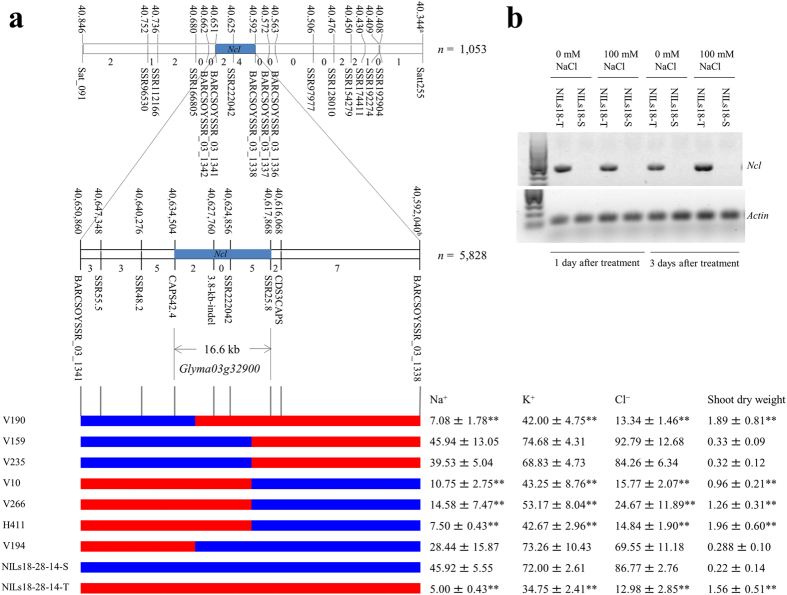
Map based cloning and expression of the salt tolerance gene *Ncl*. (**a**) Fine mapping delimits *Ncl* to a 16.6-kp region between SSR25.8 and CAPS42.4 on chromosome 3. ^a^marker position (kb), ^b^marker position (bp). Red and blue bars represent homozygous chromosome segments for tolerance and sensitivity, respectively. Na^+^, K^+^, and Cl^−^ leaf contents (*n* = 3) and shoot dry weight (*n* = 5–8) for each recombinant line after a treatment with 100 mM NaCl for approximately three weeks in a hydroponic condition. Data are presented as means ± s.d. **significant difference at *P* < 0.01 level versus NILs18-28-14-S (Dunnett’s multiple comparison test). (**b**) Expression of *Ncl* analyzed by semi-quantitative RT-PCR in the roots at one and three days after treatment with 100 mM and 0 mM (Control) NaCl in a hydroponic condition. The actin gene was used as a control.

To identify the gene that conditioned the salt tolerance QTL, we initially conducted fine mapping using 1,053 plants derived from 6 F_8_ residual heterozygous plants, and narrowed down the QTL to a 58.8-kb region between simple sequence repeat (SSR) markers BARCSOYSSR_03_1342 and BARCSOYSSR_03_1338 (Fig. 1a). However, this genomic region was not enough for identification of the causal gene underlying the QTL because there were seven predicted genes within this region based on the Glyma 1.0 soybean gene annotation database of the Williams 82 genome sequence^39^, including two genes (*Glyma03g32890* and *Glyma03g32900*) belonging to the sodium/hydrogen exchanger family. Next, a segregation population consisting of 5,828 plants derived from F_9_ residual heterozygous plants was used for screening recombination between BARCSOYSSR_03_1342 and BARCSOYSSR_03_1338. The recombination screen facilitated in the selection of 29 plants with homozygous genotype at one marker and heterozygous genotype at the other marker. The selected plants were self-pollinated for one more generation to generate fixed recombination lines. Salt tolerance evaluation and further genotyping of the recombination lines using newly developed DNA markers delimited the QTL to a 16.6-kb interval between SSR markers SSR25.8 and CAPS42.4. Only one predicted gene, *Glyma03g32900*, existed within the 16.6-kb region (Fig. 1a). RT-PCR analysis showed that the level of transcription of *Glyma03g32900* was higher in the salt-tolerant line NILs18-T than in the salt-sensitive line NILs18-S (Fig. 1b). *Glyma03g32900* was thus determined as the causal gene underlying the salt tolerance QTL, and was named as *Ncl* followed Abel (1969)^13^ (previously designated as *qNaCl3*).

### A *Ty1/copia* type retrotransposon might cause loss of function of *Ncl* in soybean

Using the 3′-Full rapid amplification of cDNA ends (RACE) and the 5′-Full RACE methods, full-length cDNAs of the tolerant and the sensitive alleles were determined from the salt-tolerant line NILs18-T and the sensitive line NILs18-S, respectively. On the basis of the positions of start and stop codons, the coding sequence (CDS) of NILs18-T was determined to be 2,436 bp in length and encodes 811 amino acid residues; the CDS of NILs18-S was 1,131 bp in length and encodes 376 amino acid residues. Comparison of genomic and cDNA sequences showed that *Ncl* harbored five CDSs in the salt-tolerant line NILs18-T, whereas three CDSs were detected in the sensitive line NILs18-S (Supplementary Fig. S1a online). The NILs18-S possessed a 3.8-kb insertion compared with the tolerant line NILs18-T. Because there were long repeated sequences within the 3.8-kb fragment, which suggested that the element belongs to a retrotransposon family, we conducted a BLAST analysis using a soybean transposable element database. We found that the 3.8-kb fragment is probably a *Ty1/copia* type retrotransposon. Alignment of the full-length cDNA sequences for NILs18-T and NILs18-S showed a point mutant at the 5′-untranslated region (UTR). In the 3′-end of the sensitive line (NILs18-S) cDNA, two polyadenylation signals of AATAAA were detected at 38 bp and 60 bp upstream of the poly (A) tail (Supplementary Fig. S2 online). The polyadenylation signal, which was introduced by the *Ty1/copia* type retrotransposon, may be responsible for the transcription termination of *Ncl* in the sensitive line.

### The genotype of the soybean root, but not shoot, determines salt tolerance

In this study, *Ncl* expression was significantly higher in the root tissues compared with the other plant parts (data not shown). To understand the role of the root system in salt tolerance, we grafted the arable part of the salt-tolerant line NILs25-T to the root of the sensitive line NILs25-S, or in reverse, to produce two kinds of graft hybrids: NILs25-T (shoot)/NILs25-S (root) and NILs25-S (shoot)/NILs25-T (root). Salt tolerance evaluation for the two kinds graft hybrids as well as the NILs pair NILs25-T and NILs25-S with 100 mM NaCl in a hydroponic condition revealed that NILs25-T and the graft hybrid NILs25-S/NILs25-T showed higher salt tolerance than NILs25-S and the graft hybrid NILs25-T/NILs25-S in terms of leaf SPAD value, shoot dry weight, and Na^+^, K^+^, and Cl^−^ concentration in the shoots, indicating that the root genotype, but not the shoot genotype, determines salt tolerance in soybean (Supplementary Fig. S3 and S4 online).

### *Ncl* synchronously regulates the accumulation of Na^+^, K^+^, and Cl^−^ in soybean leaves

BLAST analysis showed that the *Ncl* was homologous to the Na^+^/H^+^ antiporter gene family (Supplementary Fig. S1b online). It had regulatory functions for Na^+^ and K^+^ transport in soybean plant. However, our analysis observed that *Ncl* also regulated Cl^−^ accumulation. The salt-tolerant lines showed lower Na^+^, K^+^, and Cl^−^ contents in the leaves than the sensitive lines under salt stress conditions (Fig. 1a; Supplementary Fig. S4 online). Another interesting result was that the Na^+^ and K^+^ distributions in soybean plants (roots and shoots) were different from that of Cl^−^ under salt stress conditions. In comparison with the salt-sensitive lines, the salt-tolerant line accumulated lower Na^+^ and K^+^ concentrations in both shoots (leaves and stems) and roots. In contrast, the salt-tolerant line had lower Cl^−^ concentrations in shoots (leaves and stems) and higher Cl^−^ concentration in root (Supplementary Fig. S4 online). We also tested the three sets of NILs under the stress condition of 100 mM KCl. No significant differences in leaf Cl^−^ content were observed between the tolerant and sensitive lines (Supplementary Fig. S5a online). Both tolerant and sensitive lines showed chlorosis symptom under 100 mM KCl stress condition (Supplementary Fig. S5b online). *Ncl* functioned like the cation-chloride cotransporter (CCC) genes that were identified in other plants. However, *Ncl* showed a very low similarity to these putative CCC genes identified in other plant species (Supplementary Fig. S1b online).

### Overexpression of *Ncl* resulted in improved salt tolerance in transgenic soybean

**Figure 2:**
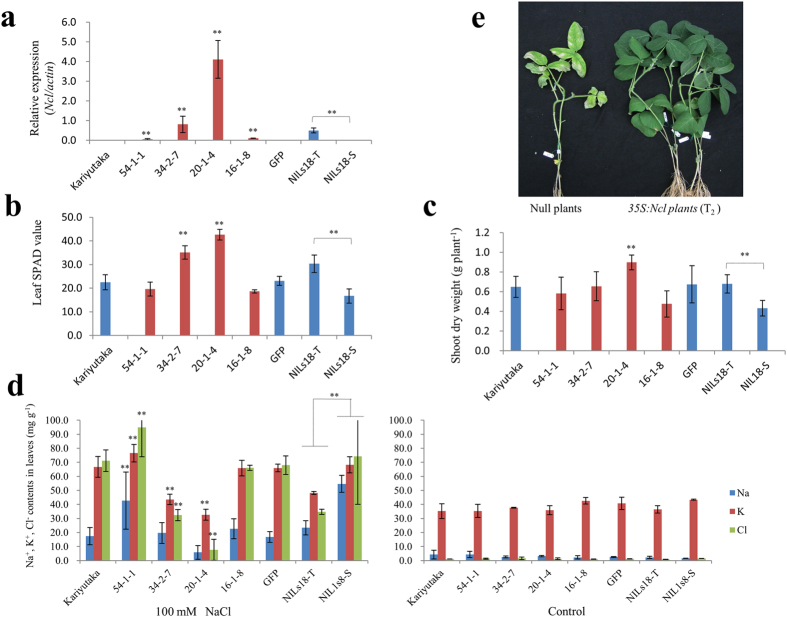
Overexpression of *Ncl* in transgenic lines enhanced salt tolerance. (**a**) Real-time quantitative RT-PCR analysis of *Ncl* expression levels in the transgenic soybean lines and control materials after treatments with 100 mM NaCl for 24 hours in a hydroponic condition. 54-1-1, 34-2-7, 20-1-4, and 16-1-8 are T_2_ 35S:*Ncl* transgenic lines. Kariyutaka: wild-type soybean cultivar. GFP is *35S:GFP* transgenic line (T_6_). NILs18-T and NILs18-S are tolerant and sensitive near isogenic lines. Values represent means from three biological replicates. (**b,c**) Leaf SPAD values and shoot dry weight for the transgenic lines after treatments with 100 mM NaCl for approximately three weeks in a hydroponic condition. (**d**) Na^+^, K^+^, and Cl^−^ leaf contents for the transgenic lines after treatments with 100 mM NaCl and 0 mM NaCl (Control) for approximately three weeks in a hydroponic condition. (**e**) Comparison of salt tolerance between *35S:Ncl* plants and null plants of the T_2_ transgenic line 20-1-4. Data are expressed as means ± s.d. **indicate significant difference at *P* < 0.01 levels versus from Kariyutaka (wild-type) (Dunnett’s multiple comparison test).

We transformed *Ncl* full-length cDNA driven with 35S promoter (*35S:Ncl*) into a Japanese soybean cultivar Kariyutaka using the *Agrobacterium*-mediated transformation method. We used Kariyutaka for gene transformation because it is highly amenable to *Agrobacterium*-mediated transformation^40^ and is a salt-sensitive cultivar with a relatively low level of expression of *Ncl* and the same gene structure as the sensitive line NILs-18S (this study). We investigated four T_2_ transgenic lines (54-1-1, 34-2-7, 20-1-4, and 16-1-8), which were derived from independent explants, for their expression of *Ncl* and salt tolerance performances. All the four T_2_ lines showed significantly higher levels of expression of *Ncl* compared with that of the wild-type Kariyutaka and a *35S:GFP* transgenic line that was used as control, either in the salt stress of 100 mM NaCl or in the control condition (Fig. 2a; Supplementary Fig. S6a online). A close association between expression of *Ncl* and salt tolerance was observed (Fig. 2b–e). The 20-1-4 line, which was single copy number for *Ncl* (Supplementary Fig. S6b online), showed the highest expression level in the four transgenic lines. As expected, the 20-1-4 line showed the highest salt tolerance in terms of leaf SPAD value, shoot dry weight, and ions (Na^+^, K^+^, and Cl^−^) concentration in leaves. The results generated by the soybean transgenic line confirmed the function of the *Ncl* gene in relation to soybean salt tolerance.

### Expression of *Ncl* was closely associated with salt tolerance in 123 soybean germplasms from different countries

To understand the function of *Ncl* on salt tolerance in different genetic backgrounds, we analyzed the relationship between *Ncl* expression level and salt tolerance in 123 soybean germplasms, including the three sets of NILs for salt tolerance and seven wild soybean accessions. The cultivated soybean germplasms were collected from nine countries (Supplementary Table S2 online). The expression levels of *Ncl* showed a close correlation with salt tolerance in terms of leaf SPAD values (*r* = 0.7214, *P* < 0.01) and STR (*r* = 0.7476, *P* < 0.01) (Supplementary Fig. S7 online), despites the fact that the mRNA level and protein level were not always associated. This result indicated that *Ncl* was effective in different genetic backgrounds. Other genes need to be considered to explain the difference of expression level of *Ncl* among the accession that harbored *Ncl*.

### Introgression of *Ncl* into the salt-sensitive cultivar Jackson by MAS

**Figure 3:**
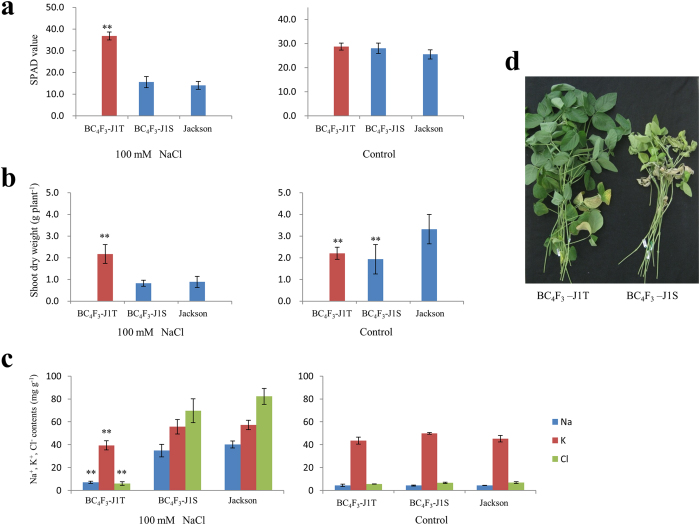
Effect of *Ncl* on salt tolerance in terms of leave SPAD value (**a**), shoot dry weight (**b**), and ion (Na^+^, K^+^, and Cl^−^ leaf contents (**c**) in BC_4_F_3_ lines developed by MAS by introducing the tolerance allele from a wild soybean accession JWS156-1 into Jackson. BC_4_F_3_-J1T and BC_4_F_3_-J1S was derived from progeny of self-pollination of a BC_4_F_2_ plant, which was heterozygous in the *Ncl* region. (**d**) Performance of BC_4_F_3_-J1T and BC_4_F_3_-J1S after treatment with 100 mM NaCl for approximately three weeks in a hydroponic condition. Data are shown as mean ± s.d. (*n* = 11). **indicate significant difference at *P* < 0.01 levels versus Jackson (Dunnett’s multiple comparison test).

To determine the usefulness of *Ncl* in soybean breeding, we conducted introgression of the salt tolerance allele identified in a wild soybean^8^ into a salt-sensitive variety, Jackson (PI 548657), through continuous backcross, followed by MAS using primers Satt339, SSR222042, and SSR112166 in each generation. A BC_4_F_2_ plant, which was heterozygous for the three SSR markers, was self-pollinated to generate a salt-tolerant homozygous line BC_4_F_3_-J1T and a sensitive homozygous line BC_4_F_3_-J1S. Salt tolerance evaluation with 100 mM NaCl in a hydroponic condition showed that BC_4_F_3_-J1T had significant higher leaf SPAD values and shoot dry weight, and lower leaf ion (Na^+^, K^+^, and Cl^−^) contents than BC_4_F_3_-J1S as well as Jackson. In contrast, no significant differences in leaf SPAD value, shoot dry weight, and leaf ion (Na^+^, K^+^, and Cl^−^) content in the control condition were observed between BC_4_F_3_-J1T and BC_4_F_3_-J1S (Fig. 3a–d). This result demonstrated that the DNA markers around *Ncl* could be used for introgression of *Ncl* into a salt-sensitive cultivar for developing of a soybean variety for high salt tolerance.

### The *Ncl* gene could increase soybean grain yield 3.6–5.5 times in saline field conditions

**Figure 4:**
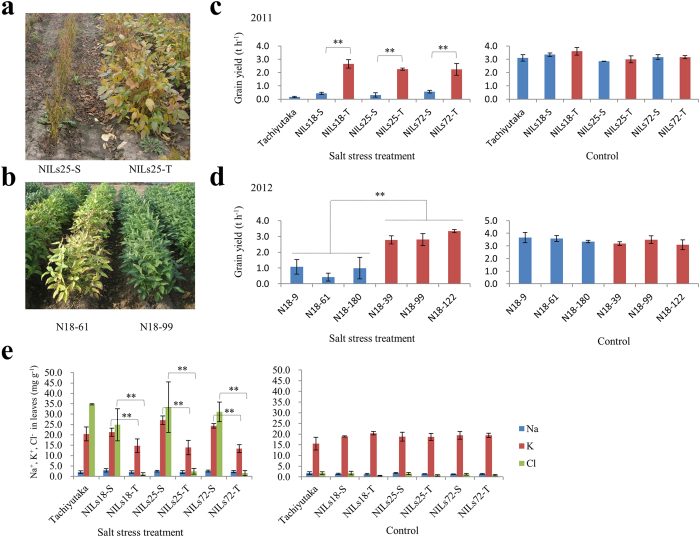
Yield performances of lines carrying *Ncl* in a saline field condition in Miyagi Prefecture, Japan. (**a**) Top view of NILs25-S and NILs25 T grown in a salt stress field in 2009. (**b**) Top view of N18-61 and N18-99 grown in a salt stress field in 2012. (**c**) Grain yield result of the three sets of NILs in 2011 field test. (**d**) Grain yield result of six recombinant lines derived from an F_8_ residual heterozygous line (RHL18-28) in 2012 field test. N18-39, N18-99, and N18-122 are lines carrying tolerant allele of *Ncl*, whereas N18-9, N18-61, N18-180 are lines that had the sensitive allele. (**e**) Na^+^, K^+^, and Cl^−^ leaf contents for the three sets of NILs in 2011 field test. Data are shown as mean ± s.d. from three replicates. **Significant difference (*P* < 0.01) based on ANOVA (Tukey’s multiple comparison test).

To determine the effect of *Ncl* in a salt stress field condition and its potential in soybean breeding for salt tolerance, we evaluated the NILs for salt tolerance in a salt stress field condition. The field experiments were conducted for three years. In 2009 and 2011, the three sets of NILs, including a salt-sensitive cultivar, Tachiyutaka, were tested. As a result, all the lines with the *Ncl* allele showed a significantly higher yield than the lines with the sensitive allele (Fig. 4a–e; Supplementary Fig. S8 online). In the 2011 field experiment, the average yield of the three tolerant lines (NILs18-T, NILs25-T, and NILs72-T) was 2.383 t/h, whereas the average yield of the three sensitive lines (NILs18-S, NILs25-S, and NILs72-S) was 0.437 t/h, increasing the grain yield by 5.5 times in a saline field condition (Fig. 4c). In 2012, we tested another six fixed lines derived from an F_8_ residual heterozygous line for the QTL region. These six lines have the same genetic background but different chromosome length for the salt tolerance QTL region. Three (N18-39, N18-99, and N18-122) of these lines were salt-tolerant, whereas three lines (N18-9, N18-61, N18-180) were salt-sensitive. The results of field experiment showed that the average yield for the three tolerant lines was 2.973 t/h, whereas that for the three sensitive lines was 0.828 t/h, indicating an increase in the grain yield by 3.6-fold in a saline field condition (Fig. 4d). In contrast, no significant differences in grain yield were observed between the tolerant and sensitive NILs in a control condition (Fig. 4c,d). Salt stress reduced the grain yield for all the lines by 55.8% in 2011 and 44.0% in 2012. For the lines with the *Ncl* allele, the yield decreased on average by 26.7% in 2011 and 29.5% in 2012, whereas those without the *Ncl* allele were 86.0% in 2011 and 76.5% in 2012. These results clearly indicated that *Ncl* contributed to achieving sustainable soybean production in a salt stress condition.

## Discussion

Previous studies on plant salt tolerance mainly focused on the toxic effects of Na^+^ or the balance of Na^+^ and K^+^ 21,22,23,24,25,26,27. Because BLAST homology search showed that *Ncl* belonged to the Na^+^/H^+^ antiporter gene family, we expected to observe Na^+^ and K^+^ regulation by *Ncl* as observed in the studies of Qi *et al.*^12^ and Guan *et al.*^20^. However, the present study not only observed this regulatory event in soybean, but also the controlled accumulation of Cl^−^. The similar results for regulation of Cl^−^ accumulation were observed in the fine-mapping, grafting, transgenesis, gene introgression by marker-assistant selection (MAS), and field testing experiments. Previous reports have described the high accumulation of Cl^−^ in soybean when subjected to salt stress^1^,^7^,^11^,^13^,^31^; however, no gene that regulates the uptake, transport, and accumulation of Cl^−^ in soybean has been identified. The CCC gene has been well studied in animals^41^,^42^; however, information on this gene in various plant species is limited. An OsCCC1 gene that regulates K^+^ and Cl^−^ but not Na^+^ was reported in rice^43^. An AtCCC gene was cloned in *Arabidopsis thaliana*, and its protein operated as a Na^+^K^+^Cl^−^ co-transporter^44^, providing evidence for the occurrence of cation-Cl^−^ cotransport in the plant kingdom. However, *Ncl* showed very low level of similarity to these putative CCC genes. Although BLAST homology search showed that *Ncl* belonged to the Na^+^/H^+^ antiporter gene family, only the first half part of *Ncl* (around 1–400 aa) showed homology with Na^+^/H^+^ antiporter gene. As for the remaining half part, no significant homologous hit was detected. The function of this region remains unclear. The fact that *Ncl* synchronously regulates the transport and accumulation of Na^+^, K^+^, and Cl^−^ suggests that it may has salt tolerance function differing from the previous salt tolerance genes identified in plant.

Transgenic line 20-4-1 showed a significantly higher *Ncl* expression compared with that of the wild type Kariyutaka as well as that of the salt-tolerant line NILs18-T. This feature resulted in the transgenic line 20-4-1 having significantly higher salt tolerance compared with those not only in the wild-type cultivar Kariyutaka but also in the tolerant line NILs18-T. This result implied that overexpression of *Ncl* may generate a higher salt tolerance line than the current available salt tolerant soybean germplasm. To determine the potential of the *Ncl* transgenic lines in practical breeding, further studies on possible negative effects in the *Ncl*-overexpressing transgenic line and its salt tolerance performance in more severe salt stress conditions are needed.

A wild soybean accession W05, from which a salt-tolerant gene (*GmCHX1*) was identified, was proposed to be used be used for soybean breeding for salt tolerance^12^. Guan *et al.* also identified the same salt-tolerant gene (*GmSALT3*) from a Chinese commercial cultivar Tiefeng 8^20^. The present study detected many wild and cultivated soybean accessions that harbored the tolerant allele of *Ncl*. Some cultivated soybean cultivars that highly express *Ncl* and show a greater degree of salt tolerance, such as S-100 and FT-Abyrara, were identified. The fact that both wild and cultivated soybeans showed variations in salt tolerance suggested that the loss-of-function mutation in *Ncl* occurred before the domestication of soybean from its ancestral wild species. The close association between *Ncl* expression and salt tolerance also demonstrated that *Ncl* is effective in different genetic backgrounds. In the present study, we transgressed the salt tolerance allele from the wild soybean accession, JWS156-1, to a cultivated soybean cultivar, Jackson. After four cycles of backcrossing, the effect of the salt tolerance gene was confirmed. However, the BC_4_F_3_ introgression lines still generated a lower biomass yield compared with its recurrent parent, Jackson, in a control condition. Therefore, the cultivated soybean germplasm may be more efficiently used in breeding cultivars with improved salt tolerance compared with that of wild soybean, which generally possesses several undesirable agronomic traits.

Evaluation of the effect of a salt tolerance gene on grain yield is extremely difficult when conducted in a nature field condition. One reason is that the concentration of salt is generally uneven both horizontally and vertically in the field. Moreover, it is influenced by several environmental factors such as rainfall. In the present study, field experiments were conducted in a paddy field, which was irrigated with diluted seawater. This method provided a uniform salt stress for each soybean line. Moreover, salt tolerance NILs were used to study the effect of the salt tolerance gene. NILs have almost the same genetic background, which in turn can rule out the influences of other traits such as flowering time, maturity, and plant size on salt evaluation, thus facilitating in determining the actual effect of the particular gene on yield. The results of the field experiments showed that the *Ncl* gene could increase soybean grain yield by 3.6–5.5-fold in a saline field condition. To confirm the general effect of *Ncl*, field tests for salt tolerance of the NILs are currently in progress in China, India, and Indonesia. The multi-environmental tests for these materials will enable us to understand the effect of *Ncl* in these regions using different concentration or kinds of salt soils such as the sodic soil. In addition, introgression of the *Ncl* into local commercial soybean varieties is presently being conducted in these countries. Isolation and characterization of *Ncl* may contribute to the sustainability of soybean production in saline areas by introducing *Ncl* into the local soybean varieties either by MAS or gene transformation methods.

## Methods

### Map-based cloning of the salt tolerance QTL

A population comprising of 1,053 plants derived from 6 F_8_ residual heterozygous plants was initially used for fine mapping of the salt tolerance QTL. These residual heterozygous plants were selected from a RIL population derived from a cross between FT-Abyara and C01^1^. They were heterozygous at the QTL region between SSR markers Satt255 and Sat_091 on chromosome 3. Sixteen plants that were homozygous at one marker and heterozygous at another marker were selected. The selected plants were self-pollinated for one more generation to produce 16 fixed recombination lines. Salt tolerance evaluation for the 16 recombination lines using 100 mM of NaCl in a hydroponic condition and DNA marker analysis determined that the QTL was located within a 58.8-kb region between SSR markers BARCSOYSSR_03_1342 and BARCSOYSSR_03_1338. To determine the causal gene conferring salt tolerance at this major QTL, a segregation population consisting of 5,828 plants derived from 32 F_9_ residual heterozygous plants was used for screening recombination between BARCSOYSSR_03_1342 and BARCSOYSSR_03_1338. From this population, 29 plants with the homozygous genotype for one marker and heterozygous for the other marker were selected. The selected plants were self-pollinated for one more generation to produce fixed recombination lines. On the basis of the soybean genome sequence information, six DNA markers (Supplementary Table S1 online) were developed between BARCSOYSSR_03_1342 and BARCSOYSSR_03_1338. Salt tolerance evaluation and further genotyping of the recombination lines with the newly developed DNA markers were used for high-resolution mapping.

### Salt tolerance evaluation using hydroponics in the greenhouse

Salt tolerance evaluation of soybean genotypes was performed using hydroponics under greenhouse conditions at the Japan International Research Center for Agricultural Sciences, Japan, according the method described previously^19^. After around three weeks of salt treatment with 100 mM of NaCl or KCl, salt tolerance rating (STR), leaf SPAD, dry weight, and ion (Na^+^, K^+^, and Cl^−^) contents were measured for each genotype. The STR scale was classified into five grades, ranging from 1 to 5 (1, plants completely dead; 2, two-third or more leaves showed chlorosis symptoms or only upside leafs survive; 3, half or less leaves showed chlorosis symptoms; 4, one-third or less leaves showed chlorosis symptoms; 5, plants bearing normal, healthy leaves). The SPAD value for each genotype was measured using a chlorophyll meter (Konica Minolta SPAD-502, Tokyo, Japan). This SPAD value is proportional to the chlorophyll content in the leaves.

### RNA isolation and expression analysis

Soybean plant tissues were collected from different soybean genotypes, frozen in liquid nitrogen, and stored at −80 °C. Total RNA was extracted using Plant RNA extraction Kit (QIAGEN, Japan), according to the manufacturer’s protocol. The first strand cDNA was synthesized using the First Strand cDNA Synthesis Kit (Toyobo, Osaka, Japan). RT-PCR was performed using QuantiTect Rev. Transcription Kit (QIAGEN, Tokyo, Japan) with primers of Glyma03g32900_CDS6-F and Glyma03g32900_CDS6-R (Table S1 online). The reaction program was 30 s at 94 °C, 30 s at 56 °C, and 1 min at 72 °C for 32 cycles, and a final extension of 7 min at 72 °C. The soybean *actin* gene was used as reference gene in the RT-PCR analysis (GenBank Acc. No. V00450). All PCR products were separated on 1.0% agarose gels and visualized with Pharos Molecular Imager (BIO-RAD, Hercules, CA, USA). Real-time PCR was performed using SsoFast^TM^ EvaGreen® Supermix (BIO-RAD, Hercules, CA, USA) with the same primers used for RT-PCR. All the reactions were performed in a Bio-Rad CFX96^TM^ machine (BIO-RAD, Hercules, CA, USA). The soybean *actin* was used as reference gene. The reaction included an initial 30 s denaturation step at 95 °C, followed by 5 s at 95 °C, and 5 s at 55 °C for 40 cycles. The PCR products were identified by melting curve analysis conducted over the range of 65–95 °C at the end of each PCR run. The 2^−ΔΔ^Ct method was used to normalize the relative gene expression data in the qPCR assay^45^.

### Obtaining of full-length cDNA sequences in salt-tolerant and -sensitive lines using Rapid amplification of cDNA ends

Salt tolerance near isogenic lines NILs18-T (tolerant) and NILs18-S (sensitive)^19^ were used to generate full-length cDNA sequences of the salt tolerance gene *Ncl*. 3′-Full Rapid amplification of cDNA ends (RACE) and 5′-Full RACE were performed to obtain 3′- and 5′-UTR using the total RNAs extracted from NILs18-T and NILs18-S, respectively. The 3′-Full RACE was conducted using a 3′-Full RACE Core Set (TaKaRa, Otsu, Japan), following the manufacturer’s protocol. Instead of using oligo (dT) primers, an adaptor primer was used to synthesize cDNA. An upstream primer was designed using the Glyma03g32900 gene as reference to amplify 3′ RACE cDNA (Table 1). Five μl of 3′ RACE-ready cDNA was used as template in a 20-μl PCR reaction. The RT-PCR conditions included 32 cycles for 30 s at 94 °C, 30 s at 56 °C, and 2.5 min at 72 °C, followed by an extension of 7 min at 72 °C. The 5′-Full RACE was performed using 5′-Full RACE Core Set (TaKaRa, Otsu, Japan), following the manufacture’s protocol except for the 5′ End-phosphorylated reversed transcription primer, 1^st^ primer pair, and 2^nd^ primer pair (Supplementary Table S1 online). 5′ RACE cDNA synthesis was performed in 15-μl cDNA synthesis reactions containing 2.5 μg of total RNA. The 1^st^ PCR reactant was diluted 10-fold and used as template in the 2^nd^ PCR reaction. The PCR conditions included 30 cycles for 30 s at 94 °C, 30 s at 56 °C, and 30 s at 72 °C, followed by an extension of 7 min at 72 °C. The PCR products from the 3′-Full RACE-PCR and 5′-Full RACE-PCR were purified from the agarose gel using Winzard SV Gel and PCR Clean-Up System (Promega Corporation, Madison, WI, USA) according to the manufacturer’s protocol. The purified DNAs were subjected to sequence analysis.

### Soybean grafting experiment

For the soybean grafting experiment, salt tolerance near isogenic lines NILs25-T (tolerant) and NILs25-S (sensitive) were used^19^. Soybean seeds were sown in vermiculite and germinated in the greenhouse with day and night temperatures at 25 °C/20 °C. One week after emergence, the plants were transferred to a hydroponic culture (half-strength Hoagland’s and Arnon solutions). When the seedlings had developed unifoliate leaves, NILs25-T and NILs25-S were grafted onto each other as scion and rootstock. This procedure resulted in two scion/rootstock combinations: NILs25-T (shoot)/NILs25-S (root) and NILs25-S (shoot)/NILs25-T (root). Ten days after grafting, 12 plants from each grafted hybrid and the two near isogenic lines NILs25-T and NILs25-S were evaluated in hydroponic culture with the half-strength Hoagland’s and Arnon solutions containing 100 mM of NaCl.

### Measurements of ion (Na^+^, K^+^, and Cl^−^) contents in soybean plants

Harvested plants were washed with deionized water. Samples were oven-dried for 3 days at 60–70 °C, and dry weight were measured. For the analysis of Na^+^, K^+^, and Cl^−^ ion contents, dried plant samples were ground using mixer mill (Millser IFM-700G, Iwatani, Japan). Na^+^and K^+^ ion contents were determined using atomic absorption spectrometry, (Z-5010, Hitachi, Japan) after dry ashing procedure and digestion with 2 N hydrochloric acid. Cl^−^ ion content was determined using the Chloride Assay Kit (BioChain, Newark, CA, USA) after 2 days of extraction with Milli-Q water at 4 °C.

### Analysis of the association between expression level of *Ncl* and salt tolerance in 123 soybean germplasms from different countries

A total of 123 soybean accessions (117 cultivated soybean accessions and six wild soybean accessions) were used to evaluate the salt tolerance and to investigate the expression level of the *Ncl* gene (Supplementary Table S2 online). Evaluation of the salt tolerance was performed using hydroponic culture in the greenhouse. RNA samples were collected from three plants from each line after 1 day of treatment with 100 mM NaCl in a hydroponic condition for real-time quantitative PCR analysis of *Ncl.*

### Generation of *Ncl* transgenic soybean

To generate transgenic soybean overexpressing *Ncl*, we transformed *Ncl* using the *Agrobacterium*-mediated transformation method into a Japanese soybean cultivar Kariyutaka. Kariyutaka was employed for gene transformation because it is highly amenable to *Agrobacterium*-mediated transformation^40^; this cultivar is also salt-sensitive, with a relatively low level of expression of *Ncl* (this study). The full-length cDNA of *Ncl* was cloned into the pMDC123 vector between the *CaMV 35S* promoter and the *AtHSP* terminator, thus obtaining the pMDC123–35S–*Ncl*–AtHSP terminator vector with the Bar gene. The transformation followed a previously described procedure^40^. Four T_2_ transgenic soybean lines (20-1-4, 54-1-3, 34-2-7, and 16-1-8) were subjected for salt tolerance evaluation with hydroponic in the green house.

### Introgression of *Ncl* into salt-sensitive cultivar by DNA MAS

A salt sensitive soybean cultivar Jackson (PI 548657) was crossed with a salt-tolerant wild soybean accession, JWS156-1, to produce F_2_ plants and subsequent backcrosses with Jackson. DNA MAS was performed using SSR markers Satt339, SSR222042, and SSR112166 in each generation (Supplementary Table S1 online). A BC_4_F_2_ plant, which was heterozygous for the SSR markers, was self-pollinated to generate a salt tolerance homozygous line BC_4_F_3-_–J1T and a sensitive homozygous line BC_4_F_3_-J1S. These two lines, which had similar genetic backgrounds but were different for the salt tolerance QTL allele, as well as the recurrent parent cultivar Jackson, were subjected to salt tolerance evaluation using hydroponic.

### Testing the effect of *Ncl* on soybean grain yield in saline field conditions

To investigate the effect of *Ncl* on soybean grain yield in response to salt stress, we grew the *Ncl* near isogenic lines in a saline field condition at the farm of Graduate School of Life Sciences, Tohoku University (38°27′ N, 141°05′ E) located in Miyagi Prefecture, Japan, for three years, namely, 2009, 2011, 2012. In 2009 and 2011, the three pairs of near isogenic lines (NILs18-T, NILs18-S, NILs25-T, NILs25-S, NILs72-T, NILs72-S), as well as a local soybean cultivar, Tachiyutaka, were tested. In 2012, we tested another six fixed-recombination lines derived from an F_8_ residual heterozygous line RHL18 for the QTL region. These six lines have the same genetic background, but had different lengths for the chromosome segment of the salt tolerance QTL region. Three (N18-39, N18-99, and N18-122) of these lines were tolerant and three lines (N18-9, N18-61, N18-180) were sensitive. The experiment was laid out in a randomized block design with three replicates. The areas of each plot were 6.7, 13.76, and 8.0 m^2^ in 2009, 2011, and 2012, respectively, with two rows in which soybean plants were plated at 20-cm intervals between hills and 80 cm between rows. Salt stress treatments were conducted at around 5 weeks after sowing by irrigated with around 1/4 concentration of seawater and normal water (Control). Leaf samples from five plants of each line were taken after four weeks of treatment for ion (Na^+^, K^+^, and Cl^−^) in 2011. After maturity, we randomly sampled 10 plants from each plots and harvested the remains to measure grain yield.

## Additional Information

**How to cite this article**: Do, T. D. *et al.* Ncl Synchronously Regulates Na^+^, K^+^, and Cl^–^ in Soybean and Greatly Increases the Grain Yield in Saline Field Conditions. *Sci. Rep.* **6**, 19147; doi: 10.1038/srep19147 (2016).

## Supplementary Material

Supplementary Information
