# Supplementary material for: Ncl Synchronously Regulates Na+, K+, and Cl− in Soybean and Greatly Increases the Grain Yield in Saline Field Conditions
Source: Sci Rep. 2016 Jan 8;6:19147. doi: 10.1038/srep19147 (PMC4705485; doi:10.1038/srep19147)
Supplement: Supplementary Information [file srep19147-s1.pdf]

Supplementary Information File

Ncl synchronously regulates Na^{+}, K^{+}, and Cl^{-} in soybean and greatly increases the grain yield in saline field conditions

Tuyen Duc Do, Huatao Chen, Hien Thi Thu Vu, Aladdin Hamwieh, Tetsuya Yamada, Tadashi Sato, Yongliang Yan, Hua Cong, Mariko Shono, Kazuhiro Suenaga & Donghe Xu*

*To whom correspondence should be addressed. E-mail: xudh@jircas.affrc.go.jp

This PDF file includes:
Supplementary Figures 1–8, Supplementary Tables 1–2, and Reference 46

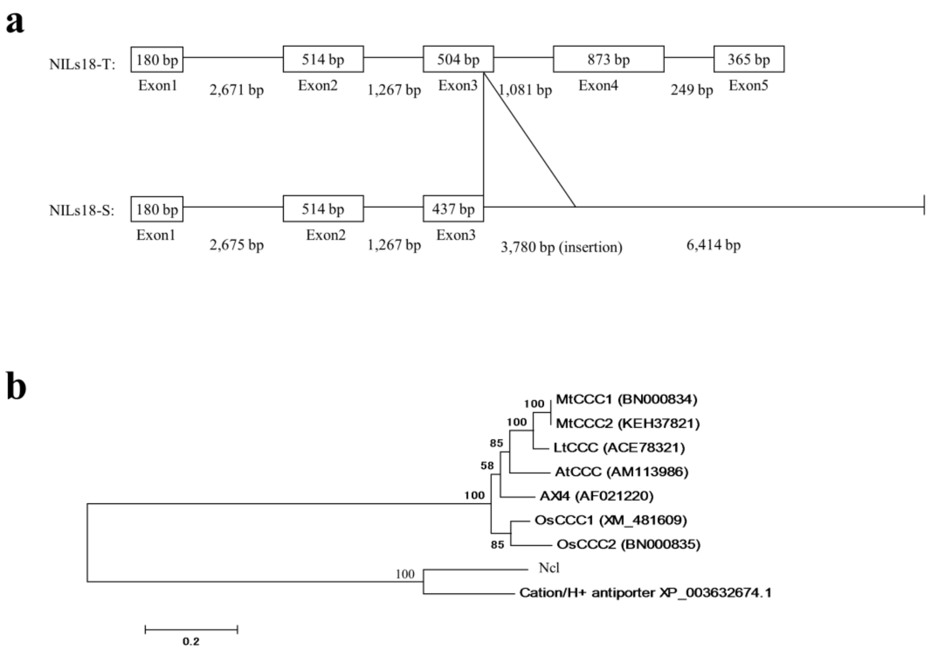

Supplementary Figure S1. *Ncl* structure and its phylogenetic relationships with other genes. (a) *Ncl* structure in tolerant (NILs18-T) and sensitive line (NILs18-S). Boxes: coding sequence; line: introns. Gene structure was determined by comparison of genomic and cDNA sequences of *Ncl* in tolerant (NILs18-T) and sensitive (NILs18-S) lines. (b) Phylogenetic tree of *Ncl* and the plant cation-chloride cotransporter genes constructed using MEGA 6.0[46] with neighbor-joining and 1,000 bootstrap replicates. Previously reported cation-chloride cotransporter sequences are retrieved from the NCBI database.

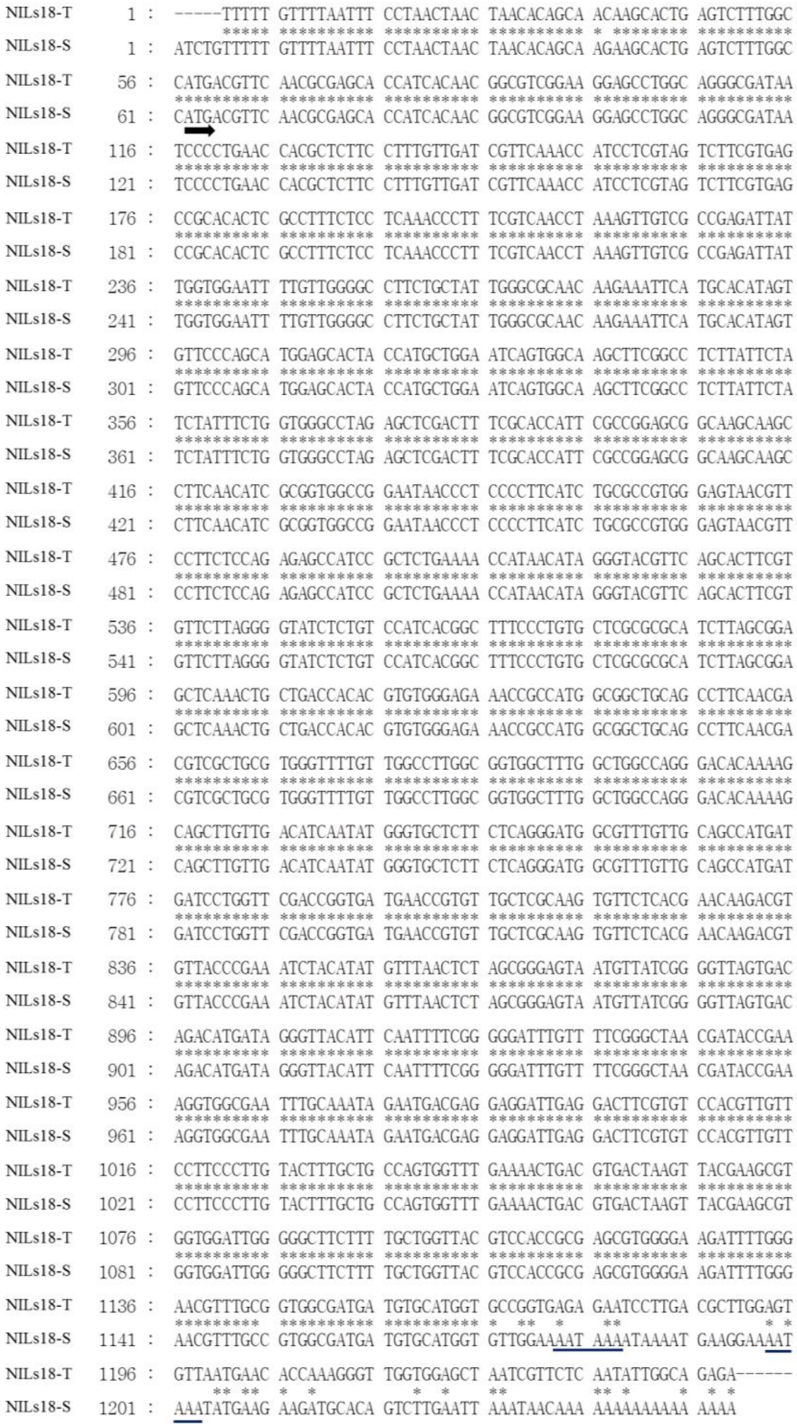

Supplementary Figure S2. Alignment of the *Ncl* cDNA sequences in tolerant (NILs18-T) and sensitive line (NILs18-S). The putative polyadenylation signal sequences in NILs18-S are underlined. The arrow indicates the start codon ATG.

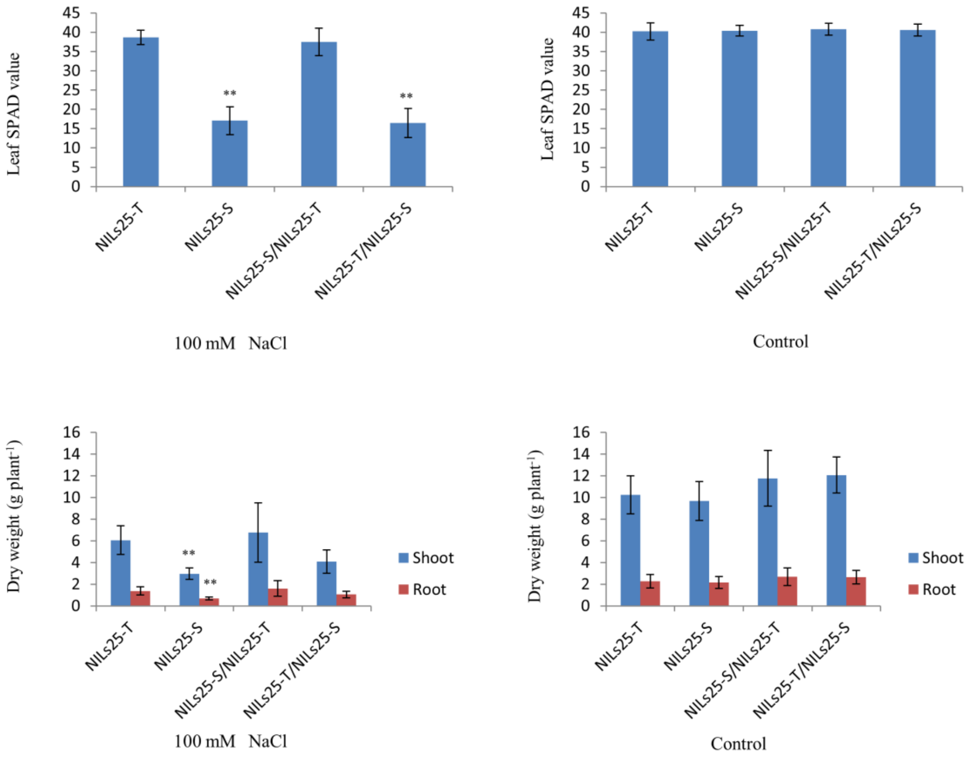

Supplementary Figure S3. Comparison of salt tolerance for salt tolerant near isogenic lines NILs25-T and NILs25-S and their graft hybrids NILs25-T/NILs25-S and NILs25-S/NILs25-T in terms of leaf SPAD value and dry weight for shoot and root. All the lines were evaluated in a hydroponic condition with 100 mM NaCl and 0 mM NaCl (Control). Data are shown as mean ± s.d. (n = 6). ** indicate significant differences versus NILs25-T at P < 0.01 level based on Dunnett's multiple comparison test.

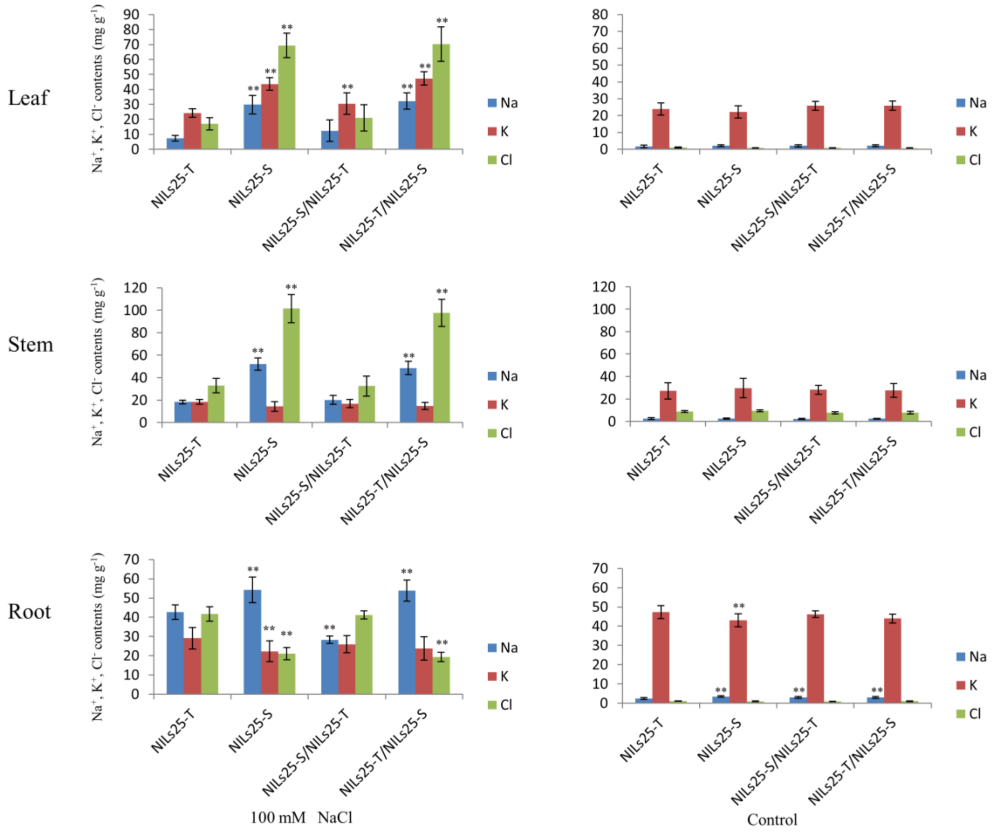

Supplementary Figure S4. Na^+, K^+, and Cl^- contents in leaves, stems, and roots in salt tolerant near isogenic lines NILs25-T and NILs25-S and their graft hybrids NILs25-T/NILs25-S and NILs25-S/NILs25-T. All the lines were evaluated in a hydroponic condition with 100 mM NaCl and 0 mM NaCl (Control). Data are shown as mean ± s.d. (n = 6). ** indicates significant differences versus NILs25-T at P < 0.01 levels based on Dunnett's multiple comparison test.

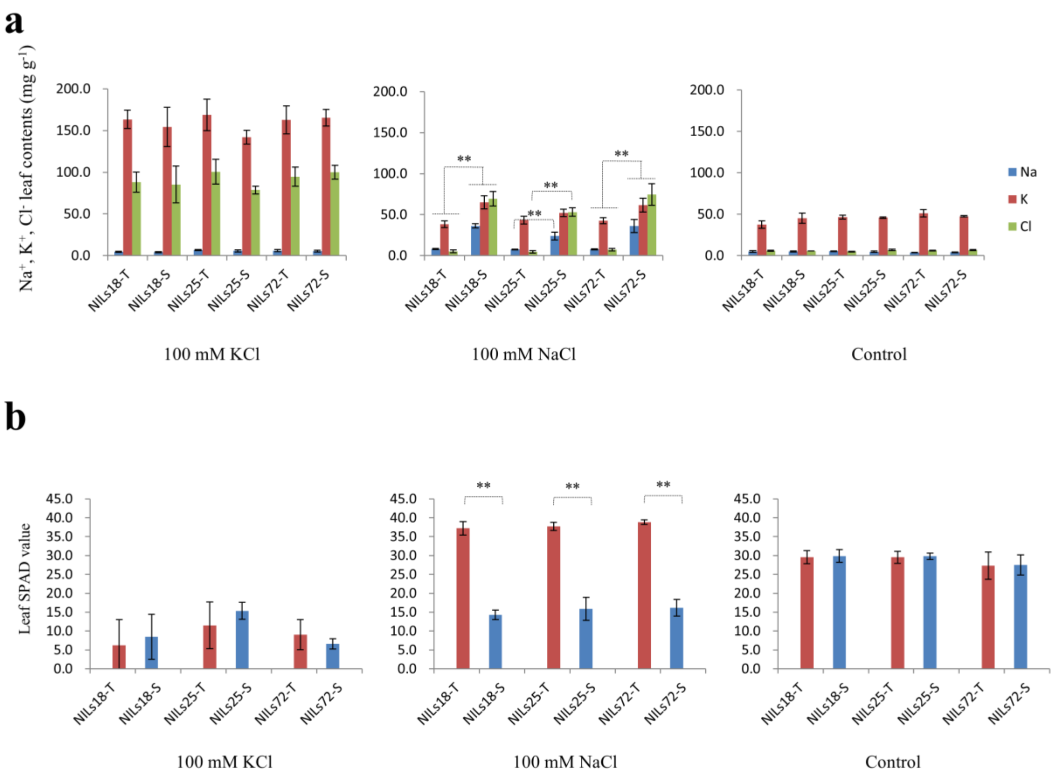

Supplementary Figure S5. Na^{+}, K^{+}, and Cl^{-} contents in leaves (a) and leaf SPAD values (b) in the three sets of salt tolerant near isogenic lines after treatment with 100 mM KCl, 100 mM NaCl, and CK for about three weeks in a hydroponic condition. Data are shown as mean ± s.d. (n = 6). **: Significant difference (P < 0.01) based on ANOVA (Tukey's multiple comparison test).

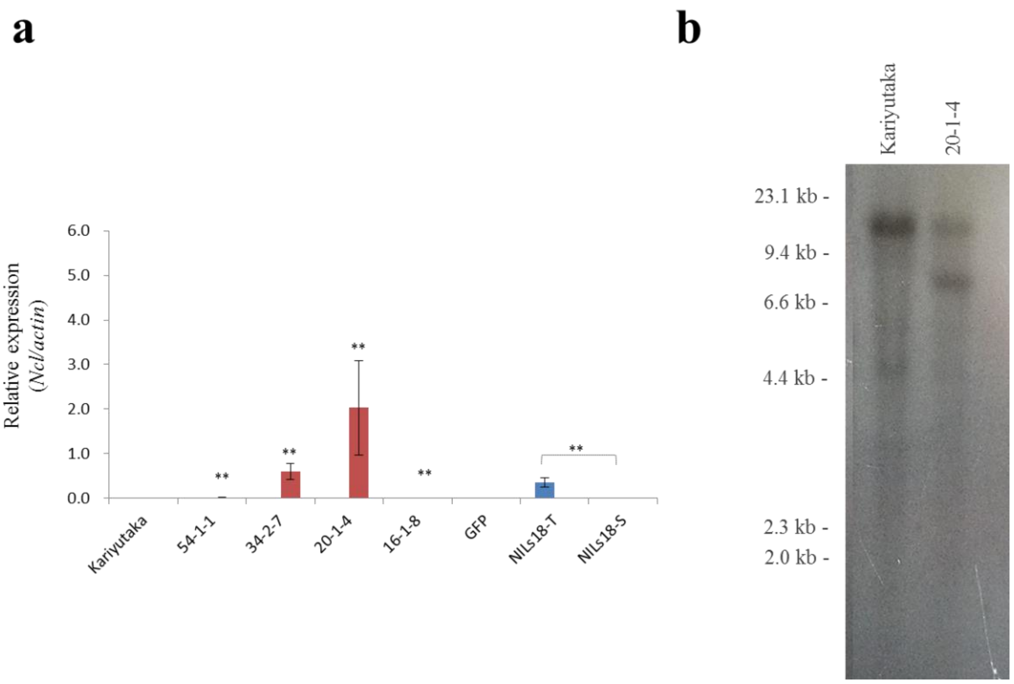

Supplementary Figure S6. Transgene expression in *35S:Ncl* transgenic lines in a control condition and Southern blot analysis of the *35S:Ncl* transgenic line 20-1-4. (a) 54-1-1, 34-2-7, 20-1-4, and 16-1-8 are T_2 35S:*Ncl* transgenic plants. Kariyutaka: wild-type soybean cultivar. GFP is *35S:GFP* transgenic line (T_6). NILs18-T and NILs18-S are tolerant and sensitive near isogenic lines. Values represent means from three biological replicates. Data are expressed as means ± s.d. ** indicates significant difference at $P < 0.01$ levels versus Kariyutaka (Dunnett's multiple comparison test). (b) Southern blot analysis of the *35S:Ncl* transgenic line 20-1-4. Genomic DNAs were digested with *XbaI*. DNA was blotted on a membrane and hybridized with Ncl gene, which has only one *XbaI* restriction site.

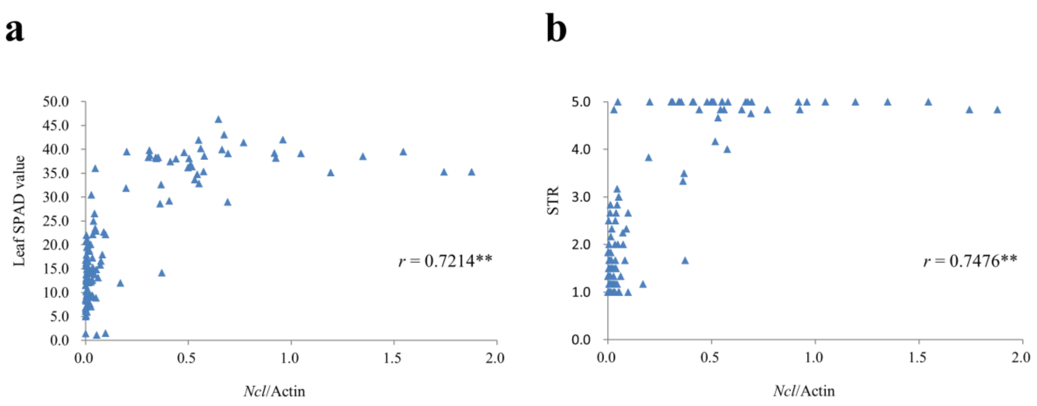

Supplementary Figure S7. Associations of expression level of *Ncl* with salt tolerance in terms of leaf SPAD value (a) and salt tolerance rating (STR) (b) in 123 soybean accessions from different countries. X-axis: real-time quantitative RT-PCR analysis of *Ncl*. Relative expression values of soybean *actin* are shown. RNA samples were collected from three plants for each line after 1 day of treatment with 100 mM NaCl in a hydroponic condition. Y-axis: leaf SPAD values and salt tolerance rating (STR). Values represent means from three different plants. Data were recorded after treatment with 100 mM of NaCl for approximately 3 weeks in a hydroponic condition. The STR scale was classified into five grades, ranging from 1 to 5 (1, plants completely dead; 2, two-thirds or more leaves showed chlorosis symptoms or only upside leafs survive; 3, half or less leaves showed chlorosis symptoms; 4, one-third or less leaves showed chlorosis symptoms; 5, plants with normal healthy leaves).

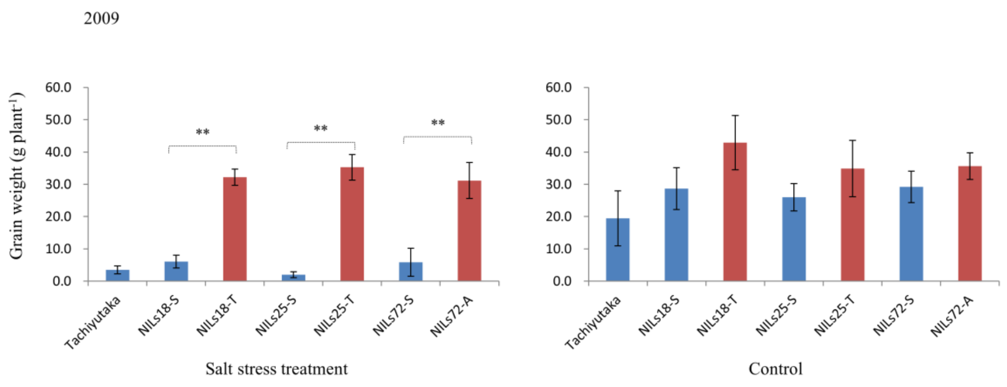

Supplementary Figure S8. Yield performances of lines carrying *Ncl* in a saline field condition in Miyagi Prefecture, Japan, in 2009. The three sets of NILs were planted in saline and control conditions. Data are shown as mean ± s.d. from three replicates. **: Significant difference (P < 0.01) based on ANOVA (Tukey's multiple comparison test).

Supplementary Table S1. Primers used in this study

Name	Sequences (5’-------3’)	Purpose
Satt255	GCGCTTTTAGCGTCGTCTGGC	Fine mapping
	TACCCCTCTCTTATTCTTCTT	
SSR192904	TCAAAACACATTTATGCAATTCG	
	CAAAATTTGATACACACAAGAGGA	
SSR192274	CATGTGCAGTCGGTAGTTCTG	
	TAGGCAATGGCCACATACAA	
SSR174411	CGGTGGTGATCGATTTTTCT	
	AATTCCGCGTGACTGAGACT	
SSR154279	CACTCATGCCAACAGTGTCA	
	GCAATTTAACGTTGAAGTCGAT	
SSR128010	CAACCAACAGGAGTTGGGTAT	
	CCTAAAGAGCCTCCTTCTCACA	
SSR97977	TGAGCCCAAATTGTGAATGA	
	AACCTCTGAAGCACGGACAT	
BARCSOYSSR_03_1336	GGCAGTGCTGTGAAAACTCA	
	AGAACCCATGACAAAAACTTGA	
BARCSOYSSR_03_1337	ACCAGCTCGTGTTGTGTTTT	
	CGCTCTCTCTATGCCTCCAC	
BARCSOYSSR_03_1338	GCATTGGTTCCTTTTCTCATGT	
	TGTTTTGACAAGGTGATTTTCA	
CDS3CAPS	CGATGAAATGTTCGCAATCTT	
	TTGGCCCAGTTGACCGTA	
SSR25.8	TTAAGCACCAGCAAATAGTTC	
	CGACCAACTATTCCCATATAC	
SSR222042	TTGAGTCTTTTCCTTTTTGAAGA	
	CGGCTCGATTTGACTTGATT	
3.8-kb-indel	CTCGCAAGTGTTCTCACGAA	
	TTAGCTCCACCAACCCTTTG	
CAPS42.4	GACATCAAGGGCCGAGAGTA	
	TGAACACGTGGTTCCTGTTT	
SSR48.2	CCTGTTAGTGGTTAATTCGAT	
	TTAAATCTTCGAATGGTTGG	
SSR55.5	TTGGATAGTAATGTTGTCTTCG	
	AAGAAAAGTGATGTGACTTGA	

BARCSOYSSR_03_1341	TTTCATATCCCTTGCCCTTG	
	CGAAATAACACCCACCATCC	
BARCSOYSSR_03_1342	GTCAACAGAACCGAGACCGT	
	ATTCACATCCTGAGCTTGGC	
SSR166805	TTGTCTTTAGGTGAAACAAACTTTTTA	
	CAAGTAACCTATCTATTTCGCACA	
SSR112166	TCCATAAATAGGTTGCCAGTTG	
	TCAAACGAGAGGAGTGTTTTGA	
SSR96530	AAGTTTCCCCCATTTCGTTC	
	TCCCTTTTCTATGAACTCCAACA	
Sat_091	CTTCTGGATAGTTGGGACTGATA	
	GGAACAGGTCGTGAAAAGTTAT	
2CDS3Up	GTTACCCGAAATCTACATATGTTTAACTCTA	3′-RACE and 5′-RACE
Ada-P	ATTCGAGACGTCTGGTACGC	
Ada-dT	ATTCGAGACGTCTGGTACGCTTTTTTTTTT	
5RACE-P	(P)GATAACATTACT	
S1	CTTCTCAGGGATGGCGTTT	
A1	ACGTGTGGTCAGCAGTTTGA	
S2	CTCGCAAGTGTTCTCACGAA	
A2	TCTCGGCGACAACTTTAGGT	
qNaClV	TCTCCACTGACGTAAGGGATG	Transgenic plant selection
	CACTGATTCCAGCATGGTAGTGC	
Glyma03g32900_GXL	TTCTTGCGACTCTTGCTCTATAAGAATACC	Genomic DNA sequencing
	CCTTTGTTTATTTCATGTGGGAGTGTATTT	
Glyma03g32900_CDS6	CCACCAACATGTCACGACTC	RT-PCR/qPCR
	ACCCCACGATTGACTAGCAC	
Glyma03g32900_CDS3	GTTTGTTGCAGCCATGATGA	
	AATCCACCACGCTTCGTAAC	
SoyAct	GTTCTCTCCTTGTATGCAAGTG	
	CCAGACTCATCATATTCACCTTTAG	
SoyActin	GAGCTATGAATTGCCTGATGG	
	CGTTTCATGAATTCCAGTAGC	
Satt339	TAATATGCTTTAAGTGGTGTGGTTATG	Marker assistant selection
	GTTAAGCAGTTCCTCTCATCACG	

SSR222042	TTGAGTCTTTTCCTTTTTGAAGA
	CGGCTCGATTTGACTTGATT
SSR112166	TCCATAAATAGGTTGCCAGTTG
	TCAAACGAGAGGAGTGTTTTGA

Supplementary Table S2. List of soybean germplasm used for studying the association between gene expression of salt tolerant gene *Ncl* and their salt tolerance

No.	Name	Acession number[a]	Species	Origin
1	947-DCE-Sj-020-1	PI222546	*G. max*	Argentina
2	FT-Abyara	PI628838	*G. max*	Brazil
3	JinDou No.6	C058	*G. max*	China
4	XinRongDaDou	C184	*G. max*	China
5	Shang Tsai	PI103079	*G. max*	China
6	Great White	PI165671	*G. max*	China
7	Pan-San	PI171437	*G. max*	China
8	No. 3032 Green	PI171439	*G. max*	China
9	No. 3093 Green	PI171440	*G. max*	China
10	Mud-bean	PI171441	*G. max*	China
11	Paoting	PI179825	*G. max*	China
12	Qi Huang No. 10	PI458512	*G. max*	China
13	Lu dou 7	PI518719	*G. max*	China
14	Yue jin 5	PI518720	*G. max*	China
15	S-100	PI548488	*G. max*	China
16	Qi huang No. 1	PI561375	*G. max*	China
17	Niu mao huang	PI567567	*G. max*	China
18	Fen dou 16	PI574476A	*G. max*	China
19	Sixth Moon	PI60273	*G. max*	China
20	7902	PI92707	*G. max*	China
21	Cibao	PI153681	*G. max*	El Salvador
22	No. 15 Seed black	PI164885	*G. max*	Guatemala
23	No. 10294	PI175198	*G. max*	India
24	No. 10296	PI175199	*G. max*	India
25	Punjab-1	PI198078	*G. max*	India
26	Local variety	PI215693	*G. max*	India
27	I-1	PI374163	*G. max*	India
28	Kedelee No. 367	PI133226	*G. max*	Indonesia
29	Ringgit	PI192867	*G. max*	Indonesia
30	Reg. No. 27	PI219652	*G. max*	Indonesia
31	Goishi Shirohana	JP27742	*G. max*	Japan
32	Fukui Shiro	JP27948	*G. max*	Japan
33	Chuusei 11	JP29316	*G. max*	Japan

34	Kariyutaka	JP86520	*G. max*	Japan
35	Chu-chou	PI157413	*G. max*	Japan
36	No. 24	PI181544	*G. max*	Japan
37	No. 42	PI181559	*G. max*	Japan
38	Aka Saya	PI200446	*G. max*	Japan
39	Aki Daizu No. 1	PI200448	*G. max*	Japan
40	Amakusa Daizu	PI200451	*G. max*	Japan
41	Amakusa Nou No. 2	PI200452	*G. max*	Japan
42	Aokimame	PI200454	*G. max*	Japan
43	Aso No. 1	PI200455	*G. max*	Japan
44	Gaku Bun	PI200466	*G. max*	Japan
45	Kiro Aki Daizu	PI200488	*G. max*	Japan
46	Shimotsu Ura	PI200525	*G. max*	Japan
47	Shira Nuhi	PI200526	*G. max*	Japan
48	Shiro Daizu No. 3	PI200530	*G. max*	Japan
49	Shiro Daizu	PI200531	*G. max*	Japan
50	Shiro Hanasaki No. 1	PI200532	*G. max*	Japan
51	Sugao Zairai	PI200538	*G. max*	Japan
52	Suzanari	PI200539	*G. max*	Japan
53	Tamana	PI200542	*G. max*	Japan
54	Tamanishiki	PI200543	*G. max*	Japan
55	Waka Shima	PI200547	*G. max*	Japan
56	Yashiro Zairai No. 1	PI200549	*G. max*	Japan
57	Yashiro Zairai No. 2	PI200550	*G. max*	Japan
58	Yonekadake	PI200551	*G. max*	Japan
59	Tookichi	PI208788	*G. max*	Japan
60	No. 10 Black Seeds	PI209577	*G. max*	Japan
61	Chasengoku	PI224269	*G. max*	Japan
62	Yamaguchi-white No. 1	PI228065	*G. max*	Japan
63	No. 3	PI230970	*G. max*	Japan
64	No. 4	PI230971	*G. max*	Japan
65	No. 6	PI230973	*G. max*	Japan
66	No. 12	PI230979	*G. max*	Japan
67	Kurosengoku	PI423886	*G. max*	Japan
68	Tousan 76	PI507414	*G. max*	Japan
69	Mammoth Yellow	PI548469	*G. max*	Japan

70	Tokyo	PI548493	*G. max*	Japan
71	Tachiyutaka	PI594289	*G. max*	Japan
72	Kurakake Daizu	PI81037	*G. max*	Japan
73	Koshoku Akidaizu	PI85897	*G. max*	Japan
74	Oyachi Senshitsadairyu	PI86113	*G. max*	Japan
75	Kiizaya	PI94159	*G. max*	Japan
76	0197	PI471938	*G. max*	Nepal
77	TGM 693	PI429328	*G. max*	Nigeria
78	Honduras	PI159924	*G. max*	Peru
79	Glycine H	PI159925	*G. max*	Peru
80	Tumbes	PI159927	*G. max*	Peru
81	No. 430	PI215755	*G. max*	Peru
82	41S31	PI159095	*G. max*	South Africa
83	Chonbuk, Sosan	K099	*G. max*	South Korea
84	White-soybean	PI157488	*G. max*	South Korea
85	No. 27 Seeds black	PI181697	*G. max*	Suriname
86	Ringgit	PI204335	*G. max*	Suriname
87	Seedcoat black	PI210178	*G. max*	Taiwan
88	Williams 82	PI518671	*G. max*	United States
89	Flyer	PI534646	*G. max*	United States
90	Hartwig	PI543795	*G. max*	United States
91	Peking	PI548402	*G. max*	United States
92	Avoyelles	PI548442	*G. max*	United States
93	Barchet	PI548443	*G. max*	United States
94	Biloxi	PI548444	*G. max*	United States
95	CNS	PI548445	*G. max*	United States
96	Cherokee	PI548447	*G. max*	United States
97	Clemson	PI548448	*G. max*	United States
98	Georgian	PI548455	*G. max*	United States
99	Otootan	PI548479	*G. max*	United States
100	Pine Dell Perfection	PI548481	*G. max*	United States
101	Rose Non Pop	PI548487	*G. max*	United States
102	Union	PI548622	*G. max*	United States
103	Hill	PI548654	*G. max*	United States
104	Forrest	PI548655	*G. max*	United States
105	Lee	PI548656	*G. max*	United States

106	Jackson	PI548657	*G. max*	United States
107	Davis	PI553039	*G. max*	United States
108	Cook	PI553045	*G. max*	United States
109	Lee 68	PI559369	*G. max*	United States
110	Young	PI633743	*G. max*	United States
111	NILs18-T	-	*G. max*	-
112	NILs18-S	-	*G. max*	-
113	NILs25-T	-	*G. max*	-
114	NILs25-S	-	*G. max*	-
115	NILs72-T	-	*G. max*	-
116	NILs72-S	-	*G. max*	-
117	JWS061-1	-	*G. soja*	Japan
118	CED98004	JP110728	*G. soja*	Japan
119	CED98010	JP110730	*G. soja*	Japan
120	CED98011	JP110731	*G. soja*	Japan
121	CED98016	JP110732	*G. soja*	Japan
122	CED97206	JP90964	*G. soja*	Japan
123	CED97210	JP90967	*G. soja*	Japan

[a]: accessions with a PI number were provide by the GRIN, USDA, USA; accessions with a JP number were provided by the NIAS Genebank, Japan; C057, C184, and K099 are provided by the Legume Base, National Bioresource Project (http://www.legumebase.brc.miyazaki-u.ac.jp/about.jsp)

Reference

46. Tamura, K., Stecher, G., Peterson, D., Filipski, A. & Kumar, S. MEGA6: Molecular Evolutionary Genetics Analysis version 6.0. *Mol. Biol. Evol.* **30**, 2725–2729 (2013).
